# Viewing Olfactory Affective Responses Through the Sniff Prism: Effect of Perceptual Dimensions and Age on Olfactomotor Responses to Odors

**DOI:** 10.3389/fpsyg.2015.01776

**Published:** 2015-11-24

**Authors:** Camille Ferdenzi, Arnaud Fournel, Marc Thévenet, Géraldine Coppin, Moustafa Bensafi

**Affiliations:** ^1^Centre National de la Recherche Scientifique UMR5292, Institut National de la Santé et de la Recherche Médicale U1028, Centre de Recherche en Neurosciences de Lyon, Université Claude Bernard Lyon 1Lyon, France; ^2^Max Planck Institute for Metabolism ResearchCologne, Germany

**Keywords:** olfaction, motor response, affect, aging, hedonics

## Abstract

Sniffing, which is the active sampling of olfactory information through the nasal cavity, is part of the olfactory percept. It is influenced by stimulus properties, affects how an odor is perceived, and is sufficient (without an odor being present) to activate the olfactory cortex. However, many aspects of the affective correlates of sniffing behavior remain unclear, in particular the modulation of volume and duration as a function of odor hedonics. The present study used a wide range of odorants with contrasted hedonic valence to test: (1) which psychophysical function best describes the relationship between sniffing characteristics and odor hedonics (e.g., linear, or polynomial); (2) whether sniffing characteristics are sensitive to more subtle variations in pleasantness than simple pleasant-unpleasant contrast; (3) how sensitive sniffing is to other perceptual dimensions of odors such as odor familiarity or edibility; and (4) whether the sniffing/hedonic valence relationship is valid in other populations than young adults, such as the elderly. Four experiments were conducted, using 16–48 odorants each, and recruiting a total of 102 participants, including a group of elderly people. Results of the four experiments were very consistent in showing that sniffing was sensitive to subtle variations in unpleasantness but not to subtle variations in pleasantness, and that, the more unpleasant the odor, the more limited the spontaneous sampling of olfactory information through the nasal cavity (smaller volume, shorter duration). This also applied, although to a lesser extent, to elderly participants. Relationships between sniffing and other perceptual dimensions (familiarity, edibility) were less clear. It was concluded that sniffing behavior might be involved in adaptive responses protecting the subject from possibly harmful substances.

## Introduction

One important characteristic of the human sense of smell is that it is a highly emotional sense. Affective responses to odors, and especially the most obvious ones such as attraction and disgust, serve important adaptive functions (Stevenson, 2010). They are involved in the regulation of behavioral response to events in the surrounding environment. Some particular smells can warn against toxic or dangerous substances (e.g., spoiled food, fire), enabling us to avoid serious environmental hazards. Other types of odor play a major role in sensory pleasure, modulating the ingestion of food, or contributing to social communication through attraction toward mates or attachment to kin. Such emotional responses to odors are expressed at different levels, from conscious and possibly verbalized subjective feelings to physiological changes and motor expression (e.g., Scherer, 2000). Measuring them thus requires differing methodological approaches, at the verbal (Churchill and Behan, 2010; Ferdenzi et al., 2013a), autonomic (e.g., Alaoui-Ismaïli et al., 1997; Bensafi et al., 2002a) and motor levels (such as sniffing behavior: Bensafi et al., 2003, 2007).

Research in animals and in humans has shown that sniffing, which is the active sampling of olfactory information through the nasal cavity, is of considerable importance in odor perception. The mere act of sniffing (whether or not an odorant is present) induces activation in the piriform cortex (Sobel et al., 1998), thus probably preparing the primary olfactory cortex for the arrival of olfactory information and detection of odors by the olfactory system. Laing, who was one of the first to investigate sniffing in humans, wrote (Laing, 1983, p. 99–102): “Perception of an [odor] in the environment usually initiates a sniffing episode […]. Each sniff appears to be of shorter duration and to have a greater inhalation velocity than a normal breath” and “this [behavior] may enhance [odor] perception by increasing the amount and rate at which [odor] molecules reach the olfactory receptor epithelium.” He also reported that sniff volume, duration and number during a sniffing episode decreased with increasing odor concentration, thus reducing the amount of inhaled odor when strong. Sniff volume and duration were also found to be inversely related to odor concentration in later studies (Warren et al., 1994; Walker et al., 2001; Johnson et al., 2003) and top-down accommodation to stimulus properties seems to occur very rapidly (160–260 ms) after onset of the first sniff (Johnson et al., 2003). This “concentration-dependent” characteristic of sniffing behavior was later exploited to set up a simple test of olfactory sensitivity based on the reduction in sniff volume and duration in presence of an odor compared to non-odorized air (Frank et al., 2003).

Although some authors have argued that other perceptual dimensions of odors such as hedonics occur too late in the neural cascade to have an influence on the nearly reflexive sniffing behavior (Johnson et al., 2003), there is now psychophysiological evidence that sniffing is modulated not only by odor intensity but also by subjective pleasantness. For example, breathed volume was visibly lower for the unpleasant odor of acetic acid than for the pleasant rose-like odor of phenylethanol (Warren et al., 1994). Similar findings were obtained comparing sniff volume in response to valeric acid compared with phenylethanol (Johnson et al., 2006), and to isointense odors of rotten egg (ammonium sulfide, unpleasant) compared with rose (phenylethanol; Bensafi et al., 2003) or strawberry (Bensafi et al., 2007), either perceived or imagined. In the latter comparison, differences extended to sniff duration, and notably, proved resilient, persisting in spite of instructions to maintain each sniff for a specific, constant duration. A pairwise comparison of groups of pleasant vs. unpleasant odorants provided similar conclusions (Prescott et al., 2010).

It is now clear that sniffing is part of the olfactory percept, since it (i) is influenced by stimulus properties, (ii) affects how an odor is perceived, and (iii) is sufficient in itself (with no odor present) to generate an olfactory percept and activate the olfactory cortex (Mainland and Sobel, 2006). However, the affective correlates of sniffing behavior, and in particular modulation of volume and duration as a function of odor hedonics, merit further investigation. Interpreting the motor expression of odor perception could, for example, be particularly informative in specific populations that are cognitively immature (children) or cognitively impaired (e.g., Alzheimer, Parkinson patients) and whose ability to verbally describe odor-related feeling is limited. However, to date many aspects of the relationship between sniffing behavior and odor hedonic valence remain unclear, in both these specific populations and the general population.

In this regard, several questions arise. Firstly, which psychophysical function best describes this relationship (e.g., linear, polynomial)? To date, only pairwise comparisons have been performed (between a pleasant and an unpleasant odor: (Warren et al., 1994; Bensafi et al., 2003, 2007; Johnson et al., 2006); or between a group of pleasant and a group of unpleasant odors: Prescott et al., 2010), which could not address this question. Secondly, does sniffing differentiate only clearly pleasant from clearly unpleasant smells, or can it discriminate between more subtle hedonic variations (e.g., slightly from strongly pleasant)? Thirdly, how sensitive is sniffing to other perceptual dimensions of odors such as familiarity or edibility? Fourthly, is the sniffing/hedonic valence relationship valid in other populations than young adults (e.g., in the elderly)? With regard to the possible use of sniffing measurement in the specific populations mentioned above, these four questions are essential and were addressed through four distinct experiments involving, for the first time, a very wide range of odorants. These aims were achieved through the use of an experimental sniffing measurement system developed in our laboratory.

## Materials and methods

### Participants

A total of 102 volunteers participated in 4 experiments (Experiment 1: 14 females, 6 males, mean age ± standard deviation = 24.45 ± 1.63 years; Experiment 2: 16 females, 6 males, 23 ± 2.71 years; Experiment 3: 14 females, 16 males, 29.40 ± 1.05 years; Experiment 4: 16 females, 14 males, 67.37 ± 0.77 years). Participants were tested individually and paid €16 for their participation. Exclusion criteria included self-reported olfactory impairment and/or neurological disease. All participants claimed normal sense of smell. The study was conducted in accordance with the Declaration of Helsinki and experimental procedures were approved by the local Lyon Sud-Est II review board.

### Odorants

Forty-eight odorants were used in Experiment 1, and 20 in Experiment 3 and 4 (19 of which were also used in Experiment 1; see Table 1). These stimuli were chosen to represent a wide range of perceived pleasantness. All odorants (molecules provided by Sigma-Aldrich) were diluted in mineral oil and presented in 15 ml flasks (opening diameter: 1.7 cm; height: 5.8 cm; filled with 5 ml solution). Stimuli were absorbed on a scentless polypropylene fabric (3 × 7 cm; 3 M, Valley, NE, USA) to optimize evaporation and air/oil partitioning.

**Table 1:** **List of the odorants used in Experiments 1, 2, 3, and 4**.

**Odorant**	**CAS number**	**Concentration (volume/volume)**	**Experiments**
(−)-Fenchone	7787-20-4	0.67	1
(+)-Fenchone	4695-62-9	0.67	1
1,8-Cineol	470-82-6	0.17	1
1-Butanol	71-36-3	0.04	1
1-Propanol	71-23-8	0.07	1
2,3-Butanedione	431-03-8	< 0.01	1
alpha-Ionone	127-41-3	29.36	1
alpha-Pinene	7785-26-4	0.1	1
alpha-Terpinene	99-86-5	0.19	1
Benzyl acetate	140-11-4	1.47	1
cis-3-Hexenylacetate	3681-71-8	0.25	1
Citral	5392-40-5	1.65	1
Citronellal	106-23-0	1.27	1
Citronellol	106-22-9	17.81	1
D-Carvone	99-49-0	1.92	1
Ethyl phenylacetate	101-97-3	4.93	1
Ethyl salicylate	118-61-6	5.48	1
Isobutyric acid	79-31-2	0.1	1
Isovaleric acid	503-74-2	0.19	1
Linalool	78-70-6	2.16	1
Myrcene	123-35-3	0.15	1
p-Cresol	106-44-5	1.84	1
Pentanol	6032-29-7	0.03	1
Propionic acid	79-09-4	0.03	1
R-(+)-limonene	5989-27-5	0.2	1
S-(-)-limonene	5989-54-8	0.2	1
Terpinen-4-ol	562-74-3	15.97	1
trans-2-Hexenylacetate	2497-18-9	0.16	1
trans-Anethole	4180-23-8	4.24	1
1-Decanol	112-30-1	33.74	1,3,4
1-Heptanol	111-70-6	0.91	1,3,4
3-Hexanol	623-37-0	0.08	1,3,4
Acetophenone	98-86-2	0.56	1,3,4
Allyl caproate	123-68-2	0.55	1,3,4
Benzaldehyde	100-52-7	0.15	1,3,4
beta-Ionone	14901-07-6	30.6	1,3,4
Dodecanal	112-54-9	27.74	1,3,4
Ethyl butyrate	105-54-4	0.01	1,3,4
Eugenol	97-53-0	13.12	1,3,4
Geraniol	106-24-1	21.26	1,3,4
Guaiacol	90-05-1	2.09	1,3,4
Heptanal	111-71-7	0.07	1,3,4
Hexanoic acid	142-62-1	3.63	1,3,4
Isoamyl acetate	123-92-2	0.03	1,3,4
Isoamyl phenylacetate	102-19-2	59.14	1,3,4
L-Carvone	99-49-0	2.37	1,3,4
Methyl anthranilate	134-20-3	12.65	1,3,4
Phenyl ethanol	60-12-8	2.66	1,3,4
Diphenyloxide	101-84-8	13.55	3,4
Beer	NA	20	2
Fig flower	NA	10	2
Flower	NA	20	2
Fruit	NA	10	2
Laundry soap	NA	1	2
Lavender flower	NA	10	2
Leather	NA	5	2
Lilac flower	NA	10	2
Magnolia flower	NA	20	2
Melon	NA	50	2
Pineapple	NA	10	2
Raspberry flower	NA	50	2
Shampoo	NA	10	2
Violet flower	NA	10	2
Wood	NA	5	2
Yogurt	NA	10	2

*NA, Not Applicable*.

In Experiment 2, 16 complex aromas were used (see Table 1). These stimuli were chosen because they represent subtle variations within the positive pole of the pleasantness scale. They were used to further investigate (after Experiment 1) the link between sniffing and pleasantness with a different, more evocative, set of odorants. All odorants (provided by Firmenich SA) were diluted in odorless dipropyleneglycol to obtain similar subjective intensities (see Delplanque et al., 2008). Solutions (4 ml) were injected into the absorbent core of cylindrical felt-tip pens (14 cm long, inner diameter 1.3 cm, Burghart, Germany).

### Sniffing measurement apparatus

**Figure 1:**
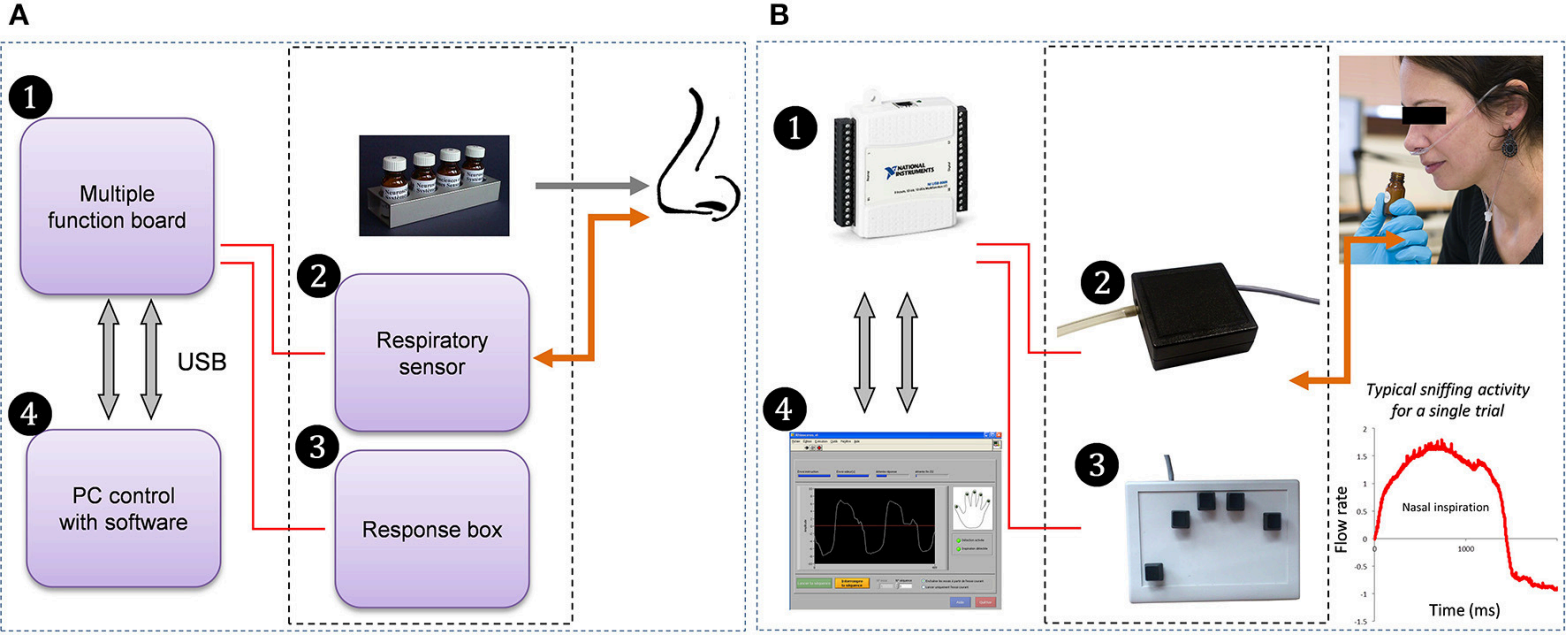
**Custom-built sniffing measurement apparatus: flow chart (A) and corresponding devices (B)**. The multiple function board (1) is used to acquire signals from the respiratory airflow sensor (2), which is connected to a nasal cannula positioned in the participant's nostril, allowing acquisition of respiratory signal, and from the 5-button response box (3) activated by the participant's fingers. The software (4) is used to set up the experimental parameters, launch the sessions and store responses.

Sniffing was recorded using a custom-built system composed of four modules (Figure 1): (1) an electronic USB device (multiple function board), (2) an airflow sensor to measure participants' nasal respiration, (3) a response box to collect subjective evaluations of odors and response times (not used in this study), and (4) dedicated software.

1. The multiple function board (National Instruments, NI-USB6009, TX, USA) was used to acquire signals from the respiratory airflow sensor and response box. It can also send output signals (Transistor-Transistor Logic: TTL) to external devices (psychophysiology or EEG recording systems, for example).
2. The airflow sensor (AWM2100V, Honeywell, MN, USA) allowed acquisition of both inhalation and exhalation phases. It was connected to a nasal cannula (Cardinal Health, OH, USA; 2.8 mm inner diameter), comprising two small tubes positioned in the participant's nostrils.
3. The custom-built response box comprises 5 buttons in a finger-wise arrangement. Box size is 178 × 127 mm. Each button is a keyboard-like switch closing a 5 V circuit.
4. The software, for the use of the experimenter, took the form of a multi-panel graphic interface. A “Participant” panel was dedicated to subject identification (participant's code and other related information) and to selecting files dedicated to implementation of the experiment. Here, all the information concerning the experimental trials and conditions (sequences of events, instructions, and questions such as olfactory dimensions to be evaluated) were read from an input ASCII file. Once the fields of this panel were filled in, the experimenter had the possibility of running an acquisition test through the “Calibration” panel, so that the respiratory signal that would be recorded during the experiment had enough amplitude without saturating. A graphic display of the signal was provided on this panel, so that the user could monitor the participant's respiratory signal in real time. Once calibration was completed, the experiment could be launched on the “Run” panel. Finally, the user could set some additional parameters and options (e.g., acquisition frequency, thresholds and scales) through the “Parameters” panel. Sniffing data, subjects' responses via the button box and related information such as response times were stored in an output ASCII results file.

### Experimental procedures

In all four experiments, participants read the instructions and provided written informed consent to the procedure before starting the experiment. Testing was performed in an experimental room designed specifically for olfactory experiments. The experimenter presented the odorants 1 cm below the subject's nose, for about 3 s. Participants were instructed to sniff at each stimulus presentation and rate hedonic valence (in all experiments), odor intensity and familiarity (in Experiments 2, 3, and 4), and edibility (in Experiments 3 and 4) on scales from 1 (not at all pleasant, intense, familiar, edible) to 9 (very pleasant, intense, familiar, edible). Odorants were presented every 20–30 s. In order to familiarize the participants with the experimental setting, they were first trained with a sequence of 1–3 non-odorized trials.

### Data analysis

For the purpose of the experiments presented here, the physiological signal was digitally recorded at 100 Hz. Sniffs were pre-processed by removing baseline offsets, and aligned in time by setting the point where the sniff entered the inspiratory phase as time zero. Maximum sniff flow rate, sniff duration and volume were calculated for the first sniff of each trial. The endpoint for volume and duration calculation was the point where the sniff returned to zero flow (end of the inspiration phase).

The relationship between hedonic ratings and sniffing behavior was analyzed with linear and degree-two polynomial regressions, with pleasantness as predictive variable and sniff characteristics as dependent variables. Where necessary, similar analyses were conducted between the other ratings (intensity, familiarity, edibility) and sniffing characteristics, and Pearson correlations were computed between pleasantness and the other ratings (intensity, familiarity, edibility). When one of these other ratings was related both to pleasantness and to a sniffing characteristic, partial correlation was conducted to determine to what extent the relationship between pleasantness and sniffing could be due to this third variable. Given the relatively large number of tests performed, it was chosen not to consider marginal effects with significance level between *p* = 0.05 and *p* = 0.10 and to give limited importance to effects with probability between *p* = 0.01 and *p* = 0.05. All statistical analyses were conducted with Statistica v.12 (Tulsa, OK, USA).

## Results

### Experiment 1 (relationship between pleasantness and sniffing for a wide range of odors, from unpleasant to pleasant)

**Figure 2:**
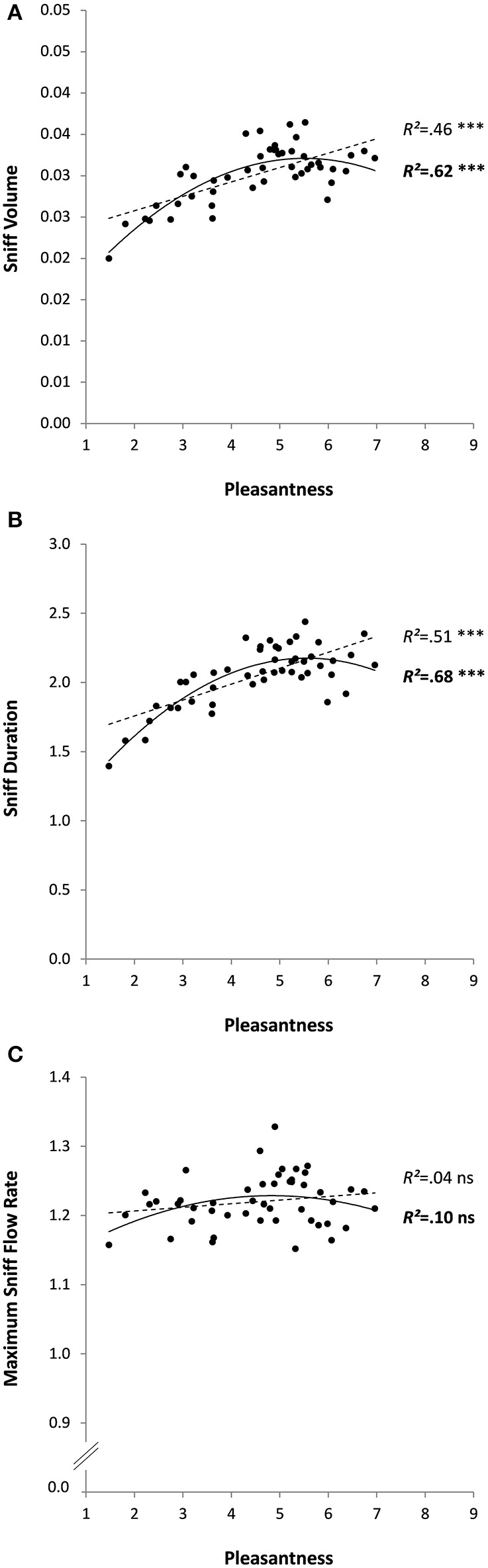
**Sniff characteristics (A: volume, B: duration, and C: maximum flow rate) as a function of odor pleasantness for 48 odorants in Experiment 1**. Linear and quadratic relationships are represented by trend curves, *R*^2^ and level of significance (^***^p < 0.001; ns: non-significant or *p* > 0.05; linear: dashed line and regular font; quadratic: continuous line and bold font).

As expected, the mean pleasantness ratings of the 48 odorants were relatively well spread out along the possible range from 1 to 9: mean pleasantness was 4.5 ± 1.4, ranging from 1.5 (for Isovaleric acid) to 7.0 (for alpha-Terpinene). Checking for outliers, defined as values greater or less than three standard deviations from the mean, found one outlier (sniff duration < M-3SD); conclusions excluding the odor in question (results in brackets) remained unchanged. There was a significant linear relationship between pleasantness and sniff volume (*R*^2^ = 0.46, *p* < 0.0001), and sniff duration (*R*^2^ = 0.51, *p* < 0.0001; without outlier: *R*^2^ = 0.45, *p* < 0.0001), but not maximum sniff flow rate (*R*^2^ = 0.04, *p* = 0.191). Coefficients were even higher when a degree-two polynomial model was used to test the relationship between pleasantness and sniff volume (*R*^2^ = 0.62, *p* < 0.0001) and between pleasantness and sniff duration (*R*^2^ = 0.68, *p* < 0.0001; without outlier: *R*^2^ = 0.60, *p* < 0.0001); the relationship between pleasantness and maximum sniff flow rate remained non-significant (*R*^2^ = 0.10, *p* = 0.100; see Figure 2). The shape of the relationship suggests that these results were due to a significant positive relationship for unpleasant odors (the more unpleasant, the smaller and shorter the sniffs), with no or maybe a converse relationship for pleasant odors. This possibility was tested by dividing the odorants into two groups: unpleasant (average pleasantness < 5; *N* = 28 molecules) and pleasant (average pleasantness >5; *N* = 20 molecules) and running the same analyses again on these subgroups. No linear regressions were significant for the pleasant odors (*R*^2^*s* < 0.23, *ps* > 0.110), whereas they were for the unpleasant odors (*R*^2^*s* >0.71, *ps* < 0.0001 for sniff volume, and for duration with or without outlier).

### Experiment 2 (relationship between pleasantness and sniffing for odors ranging from neutral to pleasant)

**Figure 3:**
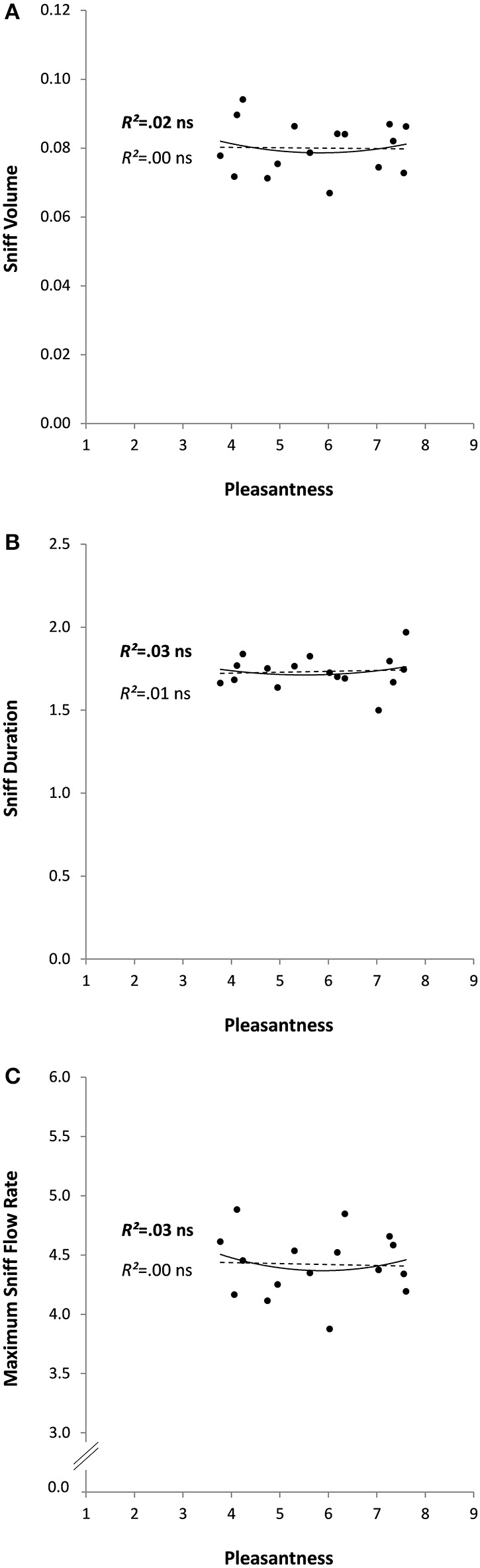
**Sniff characteristics (A: volume, B: duration, and C: maximum flow rate) as a function of odor pleasantness for 16 odorants in Experiment 2**. Linear and quadratic relationships are represented by trend curves, *R*^2^ and level of significance (ns: non-significant or *p* > 0.05; linear: dashed line and regular font; quadratic: continuous line and bold font).

In the second experiment, odors were rated as rather pleasant on average (5.8 ± 1.4, ranging from 3.8 for Leather to 7.6 for Shampoo). No outliers were found for any of the analyzed variables. In agreement with the results obtained in Experiment 1 on the pleasant sub-group of odorants, Experiment 2 found no significant relationships (linear or quadratic) between pleasantness and any of the sniff parameters (*R*^2^*s* < 0.03, *ps* > 0.110; see Figure 3 for all *R*^2^*s* and *ps*). Pleasantness was unrelated to perceived intensity (Pearson Correlation: *R* = 0.27, *p* = 0.319) and positively correlated with familiarity (*R* = 0.85, *p* < 0.0001). No significant linear or quadratic relationships were found between perceived intensity or familiarity and the sniff parameters (Table 2).

**Table 2:** **Results of the linear and quadratic regressions illustrating the prediction of sniff parameters (volume, duration, and maximum flow rate) by perceptual variables other than pleasantness (familiarity, intensity, edibility) in Experiments 2, 3, and 4**.

			**Sniff volume**		**Sniff duration**		**Maximum sniff flow rate**	
			***R*^2^**	***p***	***R*^2^**	***p***	***R*^2^**	***p***
Experiment 2	Intensity	Linear	0.06	0.343	0.00	0.925	0.13	0.174
		Quadratic	0.08	0.565	0.00	0.993	0.15	0.338
	Familiarity	Linear	0.01	0.670	0.00	0.903	0.00	0.861
		Quadratic	0.04	0.751	0.00	0.990	0.00	0.985
Experiment 3	Intensity	Linear	0.18	0.060	**0.36**	<**0.01**	0.02	0.553
		Quadratic	0.18	0.180	**0.38**	<**0.05**	0.03	0.743
	Familiarity	Linear	**0.21**	<**0.05**	0.08	0.234	0.19	0.056
		Quadratic	0.26	0.080	0.14	0.287	0.21	0.138
	Edibility	Linear	0.18	0.062	0.12	0.134	0.13	0.114
		Quadratic	0.20	0.150	0.18	0.185	0.14	0.271
Experiment 4	Intensity	Linear	0.10	0.181	**0.21**	<**0.05**	0.02	0.536
		Quadratic	0.16	0.233	0.23	0.114	0.19	0.175
	Familiarity	Linear	0.11	0.153	0.05	0.337	0.12	0.128
		Quadratic	0.11	0.363	0.13	0.321	0.13	0.297
	Edibility	Linear	**0.22**	<**0.05**	**0.21**	<**0.05**	0.14	0.110
		Quadratic	0.23	0.110	0.28	0.064	0.14	0.270

*Significant relationships (p < 0.05) are in bold*.

### Experiment 3 (relationship between several perceptual dimensions and sniffing in young adults)

**Figure 4:**
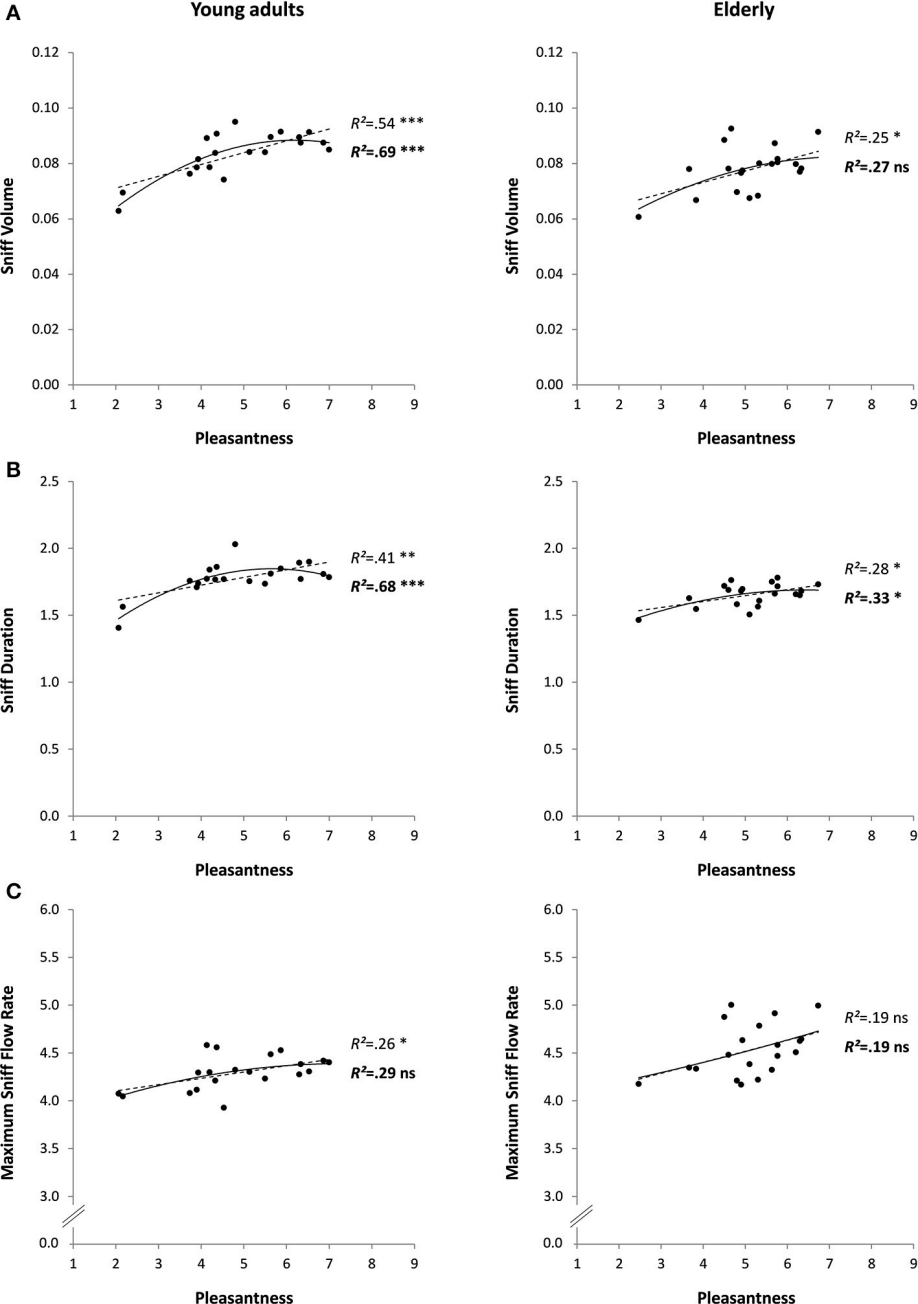
**Sniff characteristics (A: volume, B: duration, and C: maximum flow rate) as a function of odor pleasantness for 20 odorants in Experiment 3 (young adults: left column) and Experiment 4 (elderly adults: right column)**. Linear and quadratic relationships are represented by trend curves, *R*^2^ and level of significance (^***^p < 0.001; ^**^p < 0.01; ^*^p < 0.05; ns: non-significant or *p* > 0.05; linear: dashed line and regular font; quadratic: continuous line and bold font).

The 20 odorants used in this experiment were relatively varied in pleasantness: mean pleasantness was 4.9 ± 1.4, ranging from 2.1 (for Hexanoic acid) to 7.0 (for Isoamyl acetate). No outliers were found for any of the analyzed variables. The detailed results of the linear and quadratic regressions between pleasantness and sniff parameters are shown in Figure 4 (left column) and are fully in line with the conclusions of Experiment 1 on prediction of sniff volume and sniff duration by odor pleasantness. In contrast with Experiment 1, however, maximum sniff flow rate linearly increased with increasing pleasantness (*p* < 0.05). Only one relationship was significant for prediction of sniff parameters by familiarity and edibility (both of which correlated strongly with pleasantness: *R* = 0.76 and *R* = 0.84, respectively, *p* < 0.001): increasing familiarity was linearly associated with increasing sniff volume (Table 2). The partial correlation between pleasantness and sniff volume revealed a slight decrease in *R*-value and significance level (*R* = 0.67 instead of 0.73 and *p* < 0.01 instead of 0.001) when familiarity was a covariate, suggesting that familiarity is involved, although moderately, in the relationship. Again in this experiment pleasantness and intensity were independent (*R* = −0.11, *p* = 0.653), but this time intensity predicted sniff duration (significant linear and quadratic relationships, with sniffing duration decreasing with increasing intensity; Table 2).

### Experiment 4 (relationship between several perceptual dimensions and sniffing in older adults)

As in Experiment 3, the 20 odorants received relatively varied pleasantness ratings in a group of elderly participants: mean pleasantness was 5.1 ± 1.0, ranging from 2.5 (for Hexanoic acid) to 6.7 (for L-Carvone). No outliers were found for any of the analyzed variables. The detailed results of the linear and quadratic regressions between pleasantness and sniff parameters are shown in Figure 4 (right column) and are in line with the conclusions of Experiments 1 and 3 on the prediction of sniff volume and sniff duration by odor pleasantness. Although the predictions appeared to be more moderate and had lower levels of significance than in Experiment 3 with younger adults (maximum level of significance: *p* < 0.05), computation of the difference between the two age-groups' *R*s using the r-to-Fisher-z transformation revealed no significant difference (*ps* > 0.276 for the linear predictions, and *ps* > 0.104 for the quadratic predictions). When considering the prediction of sniff parameters by familiarity and edibility (both, as in Experiment 3, correlating strongly with pleasantness: *R* = 0.78 and *R* = 0.89, respectively, *p* < 0.001), only edibility was linearly associated with increasing sniff volume and duration (Table 2). Partial correlations between pleasantness and sniffing volume revealed a marked decrease in *R*-values and significance levels (sniff volume: *R* = 0.21 instead of 0.50 and *p* = 0.387 instead of < 0.05; sniff duration: *R* = 0.29 instead of 0.53 and *p* = 0.228 instead of < 0.05) when edibility was a covariate, suggesting that edibility strongly mediated the relationship. As in Experiment 3, pleasantness did not correlate with intensity (*R* = 0.18, *p* = 0.448), and intensity predicted sniff duration (significant linear relationship, with sniffing duration decreasing with increasing intensity; Table 2).

## Discussion

The aim of the series of experiments presented in this paper was to better understand the relationship between sniffing behavior and odor perceptual characteristics. Including a wide range of odorants spread over the hedonic continuum and repeating the experiment in different groups of participants allowed us not only to confirm previous conclusions that participants sniff unpleasant odors less, in volume and duration, than they do with pleasant odors (Warren et al., 1994; Bensafi et al., 2003, 2007; Johnson et al., 2006), but also to more finely describe these relationships. Especially, it was shown that (i) sniffing is sensitive to the distinction between pleasantness and unpleasantness, and to subtle variations in unpleasantness, but not in pleasantness, and (ii) the more unpleasant the odor, the smaller the spontaneous sampling of olfactory information through the nasal cavity.

Stevenson (2010, p. 14) argued that “odors are especially adept at eliciting negative emotions in humans.” In line with this, and assuming that unpleasant odors are associated with harmful substances (but see below for a discussion on this point), the present results confirmed that sniffing behavior may in some cases have adaptive value of protection against toxic substances. Firstly, sniffs of reduced duration and volume decrease the amount of inhaled odor, thus limiting the organism's exposure to a potential threat. Similar reduction of stimulus input when the stimulus is harmful has been shown in other sensory modalities (e.g., defensive responses such as blinking in response to bright light or tactile stimulation of the eye; Ongerboer de Visser, 1980). Secondly, it may also be that stimuli of high ecological value, such as unpleasant odors, are processed more quickly than stimuli with lower survival value. Top-down accommodation to stimulus properties after sniff onset (Johnson et al., 2003) may be faster when the stimulus is unpleasant, allowing adaptive behavioral response—such as initiating termination of odor sampling—to occur as soon as possible. Again, faster processing of threatening stimuli has been shown in studies in olfaction (Bensafi et al., 2002b; Jacob et al., 2003; Jacob and Wang, 2006) and other sensory modalities (e.g., emotional face processing: Calvo et al., 2006). Regarding the pleasant pole, it cannot be excluded that the ecological value of the odors we chose was not high enough to demonstrate a relationship between sniffing behavior and degree of pleasantness. Future studies should be conducted with other sets of odors including food odors with higher reward value (such as highly appetitive chocolate or vanilla, for example), and with participants in a state of hunger (a factor of great importance both in determining the current reward value of an odor—see (Small et al., 2001)—and in influencing sniffing behavior—see (Prescott et al., 2010)—but that was not controlled for in the present study); it may be that these odors will be sampled in greater amounts than more moderately pleasant odors. Our interpretation of the adaptive function of sniffing behavior should, however, be qualified, since not all unpleasant odors come from noxious sources and some harmful substances (toxic flowers such as lily of the valley or fruits such as manchineel apple) may have pleasant smells. Sniffing may constitute an early basic component of the adaptive response to smells, while higher-level processing components, occurring later in time, refine the response according to the individual's past experience and culturally shaped mental representation of the odor. For example, the sniffing response to the offensive odor of a ripe cheese may be reduced compared to a pleasant odor of, say, vanilla, but in the end the odor source will be approached and even ingested because learning has shown it to be highly appreciable.

In the light of these results, it can be hypothesized that sniffing behavior is a motor compound of the human affective processes that allows the individual to adjust to environmental conditions or events by displaying adapted behavior (Scherer, 1994; Keltner and Gross, 1999). In the olfactory modality specifically, affective responses to smells are involved in several major adaptive functions, including threat detection, ingestion and social communication (Stevenson, 2010). Some affective responses have been shown to be recurrent across cultures, which is consistent with the idea that they have an adaptive value for humans in general, independently of individual or environmental variations (Ferdenzi et al., 2013a). The present experimental setting suggested a significant involvement of olfactomotor response in at least the first function. If this is true, it should be the case for any human being, independently of individual variation such as age. And indeed it actually is the case, since we showed that the relationship between pleasantness and sniffing behavior was conserved during normal aging (Experiment 4), even though the magnitude of the effect appeared, but not significantly, to be reduced. This is consistent with a recent study comparing young and old adults, in which sniffs were larger and longer for pleasant vs. unpleasant odors, independently of age (Joussain et al., 2013), and with studies in other modalities (e.g., face perception) showing that adaptive threat detection is unimpaired in older adults (Mather and Knight, 2006).

Also in agreement with the idea that sniffing behavior might be involved in adaptive response to smells, it was shown that pleasantness predicted sniffing behavior better than other perceptual odor dimensions, such as intensity, edibility or familiarity. Although a link between intensity and sniffing parameters has been reported several times (Laing, 1983; Warren et al., 1994; Walker et al., 2001; Johnson et al., 2003), relationship was moderate in the present study, probably because intensity was not varied and odors were supposed to be comparable for intensity. Edibility and familiarity—although much more variable across odorants than intensity—only occasionally and moderately predicted sniff volume or duration in any of the four experiments. For edibility, the weakness of the link can be explained by the fact that whether an odor comes from an edible source is not the sole criterion for determining whether it is relevant to the individual and for letting it reach the nasal mucosa without restriction (e.g., social odors are highly relevant despite being non-edible; Lundström et al., 2008). Edibility was more influential in the elderly than in younger adults, suggesting that this olfactory property may be processed differently in old age (as is hedonicity, for example: Joussain et al., 2013). For familiarity, a stronger link with sniffing was expected. Indeed, novelty of an olfactory stimulus is processed even earlier than pleasantness (Delplanque et al., 2009) and it would also seem reasonable that unfamiliar (or novel) odors might induce wariness, and thus limitation of odor sampling. This was true in one instance in the present study, but familiarity and pleasantness are not a perfect match (see Ferdenzi et al., 2013b; Delplanque et al., 2015) and the latter seems to be a stronger and more reliable predictor of sniffing. In sum, pleasantness is a very prominent perceptual criterion (Engen, 1982; Yeshurun and Sobel, 2010) that individuals use to adjust their olfactomotor behavior to the environment's odorous stimulations in an adaptive fashion.

Finally, the robustness of the relationship between pleasantness and sniffing behavior could also be seen in its persistence despite variations in sniffing pattern. Sniff volume and flow rate were lower in Experiment 1 than in Experiments 2–4; this could be due to several differences between the experiments. Firstly, odors were less pleasant on average in Experiment 1 (4.5 vs. 5.8, 4.9, and 5.1 in Experiments 2, 3 and 4, respectively). Secondly, in Experiment 1 participants had to smell more than twice the number of odors presented in the other experiments, and they may have needed to protect themselves from overstimulation and subsequent sensory adaptation (Dalton, 2000), a phenomenon that makes the rating process more difficult. Thirdly, as only one judgment was performed in Experiment 1 (pleasantness) vs. 3–4 judgments in the other experiments, the amount of sensory information needed by the participants to provide their answers may have been less in Experiment 1. The fact that the prediction of sniffing volume by pleasantness was replicated in these different experiments—in spite of these behavioral differences—makes it even more significant. It is thus likely that this relationship also exists in real life, when subjects are not specifically asked to make judgments about randomly encountered odors; but this should be tested in the future with more ecological methods.

In summary, the present study offers new insights into the link between olfactomotor response and odor perception, highlighting the privileged role of hedonics in the modulation of sniffing behavior. This behavior seems, in humans of all ages, to have adaptive value in limiting the entry of potentially harmful substances into the nasal cavity. The present results suggest that sniffing measurement could be a reliable proxy for hedonic response to smells, at least for discriminating pleasant from unpleasant smells and between smells of various degrees of unpleasantness, in populations in which verbal evaluation of hedonic responses is not possible or reliable.

## Author contributions

MB designed the research; MT and GC performed data acquisition; CF, GC, AF, and MB analyzed and interpreted the data; CF and MB wrote the paper.

## Funding

This study was supported by grants from the French National Research Agency (ANR) to CF (PDOC Program, ATTRASENS Project) and to MB (EMCO program, ICEO Project).

### Conflict of interest statement

The authors declare that the research was conducted in the absence of any commercial or financial relationships that could be construed as a potential conflict of interest.

## References

[B1] Alaoui-Ismaïli O. Robin O. Rada H. Dittmar A. Vernet-Maury E. (1997). Basic emotions evoked by odorants: comparison between autonomic responses and self-evaluation. Physiol. Behav. 62, 713–720. 10.1016/S0031-9384(97)90016-0 9284489

[B2] Bensafi M. Porter J. Pouliot S. Mainland J. Johnson B. Zelano C.. (2003). Olfactomotor activity during imagery mimics that during perception. Nat. Neurosci. 6, 1142–1144. 10.1038/nn1145 14566343

[B3] Bensafi M. Rouby C. Farget V. Bertrand B. Vigouroux M. Holley A. (2002a). Psychophysiological correlates of affects in human olfaction. Neurophysiol. Clin. 32, 326–332. 10.1016/S0987-7053(02)00339-8 12490330

[B4] Bensafi M. Rouby C. Farget V. Vigouroux M. Holley A. (2002b). Asymmetry of pleasant vs. unpleasant odor processing during affective judgment in humans. Neurosci. Lett. 328, 309–313. 10.1016/S0304-3940(02)00548-7 12147332

[B5] Bensafi M. Sobel N. Khan R. M. (2007). Hedonic-specific activity in piriform cortex during odor imagery mimics that during odor perception. J. Neurophysiol. 98, 3254–3262. 10.1152/jn.00349.2007 17913994

[B6] Calvo M. G. Avero P. Lundqvist D. (2006). Facilitated detection of angry faces: initial orienting and processing efficiency. Cogn. Emot. 20, 785–811. 10.1080/02699930500465224

[B7] Churchill A. Behan J. (2010). Comparison of methods used to study consumer emotions associated with fragrance. Food Qual. Prefer. 21, 1108–1113. 10.1016/j.foodqual.2010.07.006

[B8] Dalton P. (2000). Psychophysical and behavioral characteristics of olfactory adaptation. Chem. Senses 25, 487–492. 10.1093/chemse/25.4.487 10944515

[B9] Delplanque S. Coppin G. Bloesch L. Cayeux I. Sander D. (2015). Mere exposure effect depends on an odour's initial pleasantness. Front. Emot. Sci. 6:920. 10.3389/fpsyg.2015.00920 26191021 PMC4490210

[B10] Delplanque S. Grandjean D. Chrea C. Aymard L. Cayeux I. Le Calvé B.. (2008). Emotional processing of odors: evidence for a nonlinear relation between pleasantness and familiarity evaluations. Chem. Senses 33, 469–479. 10.1093/chemse/bjn014 18403383

[B11] Delplanque S. Grandjean D. Chrea C. Coppin G. Aymard L. Cayeux I.. (2009). Sequential unfolding of novelty and pleasantness appraisals of odors: evidence from facial electromyography and autonomic reactions. Emotion 9, 316–328. 10.1037/a0015369 19485609

[B12] Engen T. (1982). The Perception of Odors. New York, NY: Academic Press.

[B13] Ferdenzi C. Delplanque S. Barbosa P. Court K. Guinard J.-X. Guo T. (2013a). Affective semantic space of scents. towards a universal scale to measure self-reported odor-related feelings. Food Qual. Prefer. 30, 128–138. 10.1016/j.foodqual.2013.04.010

[B14] Ferdenzi C. Roberts S. C. Schirmer A. Delplanque S. Cekic S. Porcherot C.. (2013b). Variability of affective responses to odors: culture, gender, and olfactory knowledge. Chem. Senses 38, 175–186. 10.1093/chemse/bjs083 23196070

[B15] Frank R. A. Dulay M. F. Gesteland R. C. (2003). Assessment of the Sniff magnitude test as a clinical test of olfactory function. Physiol. Behav. 78, 195–204. 10.1016/S0031-9384(02)00965-4 12576116

[B16] Jacob T. J. C. Fraser C. Wang L. Walker V. O'Connor S. (2003). Psychophysical evaluation of responses to pleasant and mal-odour stimulation in human subjects; adaptation, dose response and gender differences. Int. J. Psychophysiol. 48, 67–80. 10.1016/S0167-8760(03)00020-5 12694902

[B17] Jacob T. J. C. Wang L. (2006). A new method for measuring reaction times for odour detection at iso-intensity: comparison between an unpleasant and pleasant odour. Physiol. Behav. 87, 500–505. 10.1016/j.physbeh.2005.11.018 16469339

[B18] Johnson B. N. Mainland J. D. Sobel N. (2003). Rapid olfactory processing implicates subcortical control of an olfactomotor system. J. Neurophysiol. 90, 1084–1094. 10.1152/jn.00115.2003 12711718

[B19] Johnson B. N. Russell C. Khan R. M. Sobel N. (2006). A comparison of methods for sniff measurement concurrent with olfactory tasks in humans. Chem. Senses 31, 795–806. 10.1093/chemse/bjl021 16914503

[B20] Joussain P. Thevenet M. Rouby C. Bensafi M. (2013). Effect of aging on hedonic appreciation of pleasant and unpleasant odors. PloS ONE 8:e61376. 10.1371/journal.pone.0061376 23637821 PMC3634785

[B21] Keltner D. Gross J. J. (1999). Functional accounts of emotions. Cogn. Emot. 13, 467–480. 10.1080/026999399379140

[B22] Laing D. G. (1983). Natural sniffing gives optimum odour perception for humans. Perception 12, 99–117. 10.1068/p120099 6657430

[B23] Lundström J. N. Boyle J. A. Zatorre R. J. Jones-Gotman M. (2008). Functional neuronal processing of body odors differs from that of similar common odors. Cereb. Cortex 18, 1466–1474. 10.1093/cercor/bhm178 17934190

[B24] Mainland J. Sobel N. (2006). The sniff is part of the olfactory percept. Chem. Senses 31, 181–196. 10.1093/chemse/bjj012 16339268

[B25] Mather M. Knight M. R. (2006). Angry faces get noticed quickly: threat detection is not impaired among older adults. J. Gerontol. B 61, P54–P57. 10.1093/geronb/61.1.p54 16399942

[B26] Ongerboer de Visser B. W. (1980). The corneal reflex - Electro-physiological and anatomical data in man. Prog. Neurobiol. 15, 71–83. 10.1016/0301-0082(80)90016-7 7422875

[B27] Prescott J. Burns J. Frank R. A. (2010). Influence of odor hedonics, food-relatedness, and motivational state on human sniffing. Chemosens. Percept. 3, 85–90. 10.1007/s12078-010-9073-1

[B28] Scherer K. R. (1994). Emotion serves to decouple stimulus and response, in The Nature of Emotion: Fundamental Questions, eds Ekman P. Davidson R. (New York, NY: Oxford University Press), 127–130.

[B29] Scherer K. R. (2000). Emotion, in Introduction to Social Psychology: A European Perspective, eds Hewstone M. Stroebe W. (Oxford: Blackwell), 151–191.

[B30] Small D. M. Zatorre R. J. Dagher A. Evans A. C. Jones-Gotman M. (2001). Changes in brain activity related to eating chocolate: from pleasure to aversion. Brain J. Neurol. 124, 1720–1733. 10.1093/brain/124.9.1720 11522575

[B31] Sobel N. Prabhakaran V. Desmond J. E. Glover G. H. Goode R. L. Sullivan E. V.. (1998). Sniffing and smelling: separate subsystems in the human olfactory cortex. Nature 392, 282–286. 10.1038/32654 9521322

[B32] Stevenson R. J. (2010). An initial evaluation of the functions of human olfaction. Chem. Senses 35, 3–20. 10.1093/chemse/bjp083 19942579

[B33] Walker J. C. Kendal-Reed M. Hall S. B. Morgan W. T. Polyakov V. V. Lutz R. W. (2001). Human responses to propionic acid. II. Quantification of breathing responses and their relationship to perception. Chem. Senses 26, 351–358. 10.1093/chemse/26.4.351 11369670

[B34] Warren D. W. Walker J. C. Drake A. F. Lutz R. W. (1994). Effects of odorants and irritants on respiratory behavior. Laryngoscope 104, 623–626. 10.1002/lary.5541040517 7514705

[B35] Yeshurun Y. Sobel N. (2010). An odor is not worth a thousand words: from multidimensional odors to unidimensional odor objects. Annu. Rev. Psychol. 61, 219–241, C1–C5. 10.1146/annurev.psych.60.110707.163639 19958179

